# Synthesis and Characterization of Polycaprolactone Modified Trimellitate Nano-Lubricant

**DOI:** 10.3390/ma12142273

**Published:** 2019-07-15

**Authors:** Shuzhe Guan, Xuanchi Liu, Yagang Zhang, Yumei Liu, Lulu Wang, Yanxia Liu

**Affiliations:** 1 College of Chemistry and Chemical Engineering, Xinjiang University, Urumqi 830046, China; 2 Department of Chemical and Environmental Engineering, Xinjiang Institute of Engineering, Urumqi 830091, China; 3 Xinjiang Technical Institute of Physics and Chemistry, Chinese Academy of Sciences, Urumqi 830011, China

**Keywords:** TMT, viscosity index, PCL, nano-TiO_2_, nano-lubricant

## Abstract

The application of trimellitate (TMT) in the lubricating oil industry was seriously restricted because of its low viscosity index. In the work reported here, polycaprolactone (PCL) soft chain was embedded into the structure of TMT in order to improve the viscosity index. Characterization of the polymers was done by proton nuclear magnetic resonance (^1^H-NMR), Fourier transform infrared spectroscopy (FT-IR) and thermogravimetric analysis (TG). Results supported our design and were consistent with the target product structure. Performance of the prepared materials was evaluated by standard ASTM methods. Noticeably, the viscosity index of the modified TMT increased from 8 to above 100, which greatly improved its viscosity-temperature performance. As the initiator, tetrabutyl titanate (TBT) can not only complete the ring-opening polymerization of caprolactam (ε-CL) at room temperature, but also generate nano-TiO_2_ by-products with excellent anti-wear properties during the synthesis. Characterization of the nano-TiO_2_ was done by scanning electron microscopy (SEM), FT-IR, TG and X-ray diffractometry (XRD). The friction and wear tests were conducted on a four-ball friction tester and the surface morphologies of worn surfaces were investigated by SEM. The experimental results clearly showed that the modified TMT showed better viscosity index and thermal stability as compared to the unmodified one. The modified nano-TMT base oil features excellent lubricant performance with good viscosity–temperature properties, thermal stability and anti-wear properties.

## 1. Introduction

The ester groups of ester oil molecules are polar groups, which can easily adsorb on the friction surface to form a boundary oil film with excellent shear resistance [1,2]. Ester oils have been widely used in many fields due to their excellent viscous-temperature properties, thermal oxidation stability and low volatility. Currently, ester oils are used as almost all the jet engine lubricants in the world [3,4]. Furthermore, ester oil has excellent biodegradability, low toxicity and reproducibility, which can readily meet current requirements for environmental protection [5,6]. It is also one of the important reasons why the development of ester oils has received extensive attention. With the overcapacity of international trimellitic anhydride (TMA) production, high value-added products are in urgent need of development [7,8]. As a lubricating base oil, the performance of trimellitate (TMT) is similar to that of other triesters, but it suffers of a low viscosity index and poor biodegradability due to the presence of a benzene ring, which greatly limits the use of TMT in the lubricants industry [9,10]. Few methods for synthesizing and modifying the TMT base oil with excellent performance have been reported. Theoretically, the number, length and structure of ester groups have great influence on the viscosity and viscosity index of ester base oils [11,12]. Along this line, we aim to embed polyester into the structure of trimellitate base oil in order to improve the low viscosity index of base oil [13].

PCL is one kind of polymer material with excellent biodegradability, which could be obtained by ring-opening polymerization of ε-CL by initiator [14,15,16]. PCL has good stability performance, which could provide excellent performance for composites [17]. It has been widely used in the plastics industry [18,19]. As a soft segment, PCL can improve the elastic properties of the polymer. However, PCL is also a semi-crystalline material, when embedding it into the molecular structure of base oil, the low temperature performance may cause adverse effects [20]. Therefore, it is very important to study the influence of the length of the embedded chain on the performance of base oil, whether from the performance optimization or from the economic point of view.

TBT is a common raw material for the preparation of nano-TiO_2_ by the sol-gel method. It has been widely used in the field of photocatalysis [21]. As an initiator with high activity, TBT has the advantages of low cost and easy availability, which can initiate ε-CL ring-opening polymerization at room temperature or even lower temperature [22,23]. It has the characteristics of simple synthesis process, high efficiency and energy saving [24]. The literature has been reported that the Ti–O bond attached to the star-shaped poly(ε-caorolactone) (4-PCL) can maintain a certain stability in water compared with the initiator of TBT, but it is still easy to hydrolyze under acidic conditions to produce linear poly(ε-caorolactone) (L-PCL) and TiO_2_ [25]. This phenomenon is encouraging news for the target products of L-PCL and nano-TiO_2_ to synthesis modified nano-TMT base oil.

In the work reported here, 4-PCL was synthesized by ring-opening polymerization of CL at room temperature using TBT as the initiator. L-PCL and by-product of nano-TiO_2_ were obtained by hydrolysis in HCI solution. Then the polymer intermediates were synthesized by reaction of TMA with L-PCL. After that, the modified TMT base oil was obtained through esterification reaction of polymer intermediates with isobutanol. Finally, modified nano-TiO_2_ TMT base oil with excellent performance was obtained by mixing the base oil with by-product of nano-TiO_2_ in a certain proportion.

## 2. Materials and Methods

### 2.1. Materials

TMA was purchased from Baichuan chem Co., Ltd. (Wuxi, China) with Industrial grade. TBT with analytical reagent grade was obtained from Aladdin (Shanghai, China) and used directly. ε-CL was purchased from AR Shanghai Tian Scientific Co., Ltd. (Shanghai, China) with industrial grade. Organic solvents such as HCl, isobutanol, dichloromethane, ethanol and the catalyst of stannic oxide were purchased from Beilianchem Co., Ltd. (Tianjin, China) with AR grade. Commercially available nano-TiO_2_ (99% purity) was purchased from Chaowei Nano Technology Co. Ltd. (Shanghai, China), which had an average particle size distribution range of 20–30 nm and density of 0.45 g cm^−1^ (from suppliers’ data). All reagents were used directly without further treatment.

### 2.2. Schematic Illustration of Synthesizing Modified TMT Nano-Lubricant

**Scheme 1:**
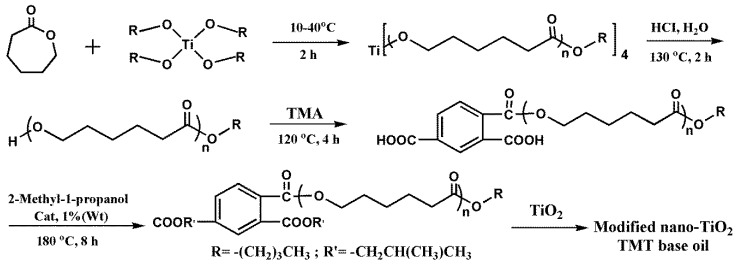
Schematic illustration of synthesizing modified TMT nano-lubricant.

The synthesis of 4-PCL was carried out by following a procedure previously described elsewhere. 16 mol ε-CL and 1 mol TBT were put into a weight three-necked round bottom flask (250 mL) equipped with a stir stick, water condenser and a glass stopper. The 4-PCL was synthesized at 25 °C and 60 rpm/min for 4 h. The crude 4-PCL was recorded using IR, TG and ^1^H-NMR. Then, 40 mL HCl solution (10 *v*/*v* %) was added into the crude product of 4-PCL, and the mixtures were stirred at 130 °C until the white precipitates were completely deposited. Then, the reaction mixture was moved into a separating funnel and extracted with CH_2_Cl_2_ (20 mL) when the system was cooled down to reach room temperature. The HCl solution layer with nano-TiO_2_ was washed several times with alcohol and distilled water to remove the residual L-PCL and HCl, then the nano-TiO_2_ was obtained by filtration and drying over at 60 °C for 24 h. The solid product was analyzed by FT-IR, TG, XRD and SEM. The organic layer was washed several times with CH_2_Cl_2_ and distilled water until a neutral pH was reached, then added into a three-necked round bottom flask. CH_2_Cl_2_ solution was removed under reduced pressure using a circulating water vacuum device at 40 °C. The L-PCL was obtained by drying over at 60 °C for 24 h, which was analyzed by FT-IR, TG and ^1^H-NMR. When the feed molar ratios of TMA/L-PCL was 1.1:1, the intermediate product of the embedded alcohol ester was obtained under the condition of 120 °C for 4 h. The vacuum distillation device was replaced with a water separator and condensing unit, excessive isobutanol (50 mL) and 1% (wt) of solid acid catalyst were added into the system. Esterification reaction was carried out for 8 h at 180 °C, and isobutanol was also used as water carrying agent. After the reaction completed, the residual isobutanol was removed by reducing pressure using a circulating water vacuum device at 100 °C. Then, the catalyst was moved by filtration. Finally, the nano-lubricant base oil with excellent performance was obtained by mixing with a certain amount of solid product of nano-TiO_2_. The experimental process was shown in Scheme 1.

### 2.3. Characterization

The infrared spectra of 4-PCL, L-PCL, TiO_2_ and the product of base oil were recorded on a DIGILAB FTS 3000 Fourier transform-infrared spectroscopy (FT-IR) spectrophotometer (Digilab Inc., Boston, MA, USA) with a KBr pellet in the range of 400 to 4000 cm^−1^ at room temperature. ^1^H-NMR spectra were obtained on a 400 MHz AVANVE 400FT-NMR spectrometer (BRUKER, Karlsruhe, Germany) with CDCL_3_ as solvent. The morphologies of TiO_2_ were imaged using a scanning electron microscope (SEM) on a JSM-5600 LV scanning electron microscope (Japan Electron Optics Laboratory Co., Ltd. Tokyo, Japan) with an acceleration voltage of 20 KV. Thermogravimetric analysis (TG) was carried out in a Perkin-Elmer TGA-7 analyzer PE TG/DTA 6300 instrument (Perkin-Elmer Inc., Boston, MA, USA) over a temperature range of 40–800 °C at an air flow rate of 50 mL/min under a heating rate of 10 °C /min. The powder X-ray diffraction (XRD) patterns of the TiO_2_ were carried out on an XRD analyzer (D8-Advance, BRUKER AXS, Karlsruhe, Germany) equipped with a diffracted-beam monochromator with Cu Ka radiation (50 kV, 40 mA). The range of 2θ was taken from 10 to 70° with steps of 0.05°. ESI-MS spectra were made on a Waters UPLC-Quattro Premier XE UPLC−MS/MS (Ultimate 3000/Q-Exactive, Thermo Fisher Scientific., Waltham, MA, USA). The ESI flow rate of the electrospray ion source was 1–2000 μL/min.

### 2.4. Performance Testing

Kinematic viscosity was conducted on a JSR1104 model Kinematic viscosimeter (JinShi PETROCHEMICAL INSTRUMENT Co., Ltd. Jinshi, China) by standards of ASTM D445 method (Standard Test Method for Kinematic Viscosity of Transparent and Opaque Liquids). Viscosity Index was measured on the standards of ASTM D2270 (Standard Practice for Calculating Viscosity Index from Kinematic Viscosity at 40 °C and 100 °C). Pour point was measured by standards of ASTM D97 method (Standard Test Method for Pour Point of Petroleum Products), which was conducted on a JSR0805 model Inclinometer (JinShi PETROCHEMICAL INSTRUMENT Co., Ltd. Jinshi, China). Acid number was measured by standards of GB/T264 method (determination of acid number, China) and the tests were carried out with KOH standard solution titration. The friction and wear tests were conducted on a four-ball friction tester (Xiamen TENKEY Co., Ltd. Xiamen, China). The test balls had a diameter of 12.7 mm, which were made of AISI 52100 steel with hardness varying from 59–61 HRC.

## 3. Results

### 3.1. ^1^H-NMR Spectra of PCL

As an initiator, TBT has been proved to initiate the ring opening of the ε-CL efficiently and rapidly at low temperatures. Then a 4-PCL was obtained by following a reported procedure [26]. The polymer could still maintain a stable four-arm star structure in the aqueous phase. However, the Ti–O bond was liable to break in the presence of HCI, which led to the hydrolysis of L-PCL and nano-TiO_2_.

**Figure 1:**
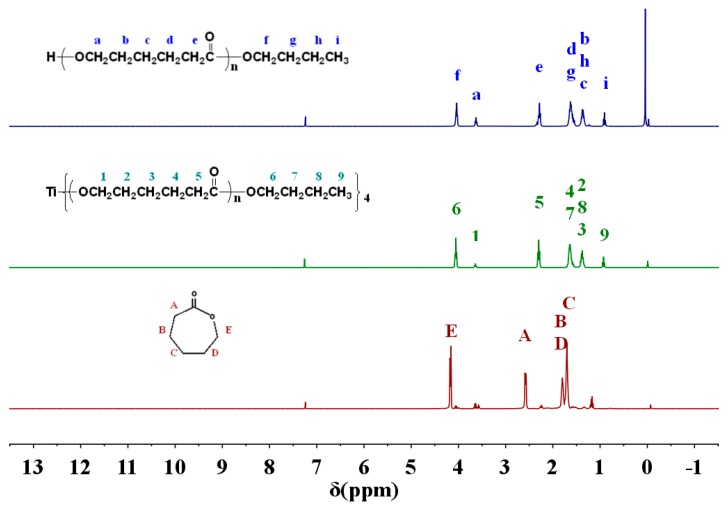
^1^H-NMR spectra of ε-CL, 4-PCL and L-PC.

ε-CL, 4-PCL and L-PCL were characterized by ^1^H-NMR analysis. A typical ^1^H-NMR spectrum of ε-CL was at δ: 4.16(t, 2H), 2.23(t, 2H), 1.80(m, 2H), 1.71(m, 2H) and 1.55(m, 2H). Figure 1 also showed that the typical 4-PCL signals of main chain unit were at δ 4.05(t, 2H), 3.61(t, 2H), 2.30(t, 2H), 1.64(m, 2H), 1.58(m, 2H), 1.41(m, 2H), 1.38 (m, 2H) and 1.33(m, 2H), while the signal at δ: 3.61(t, 2H) was assigned to the proton of –CH_2_, which was directly connected to the Ti–O bond, and δ: 0.93(t, 3H) assigned to the proton on –CH_3_ methyl group [26]. The characteristic ^1^H peaks of protons in the L-PCL block could be observed at δ: 4.04(t, 2H), 3.62(t, 2H), 2.28(t, 2H), 1.63(m, 2H), 1.58(m, 2H), 1.38(m, 2H), 1.36(t, 2H), 1.23(m, 2H) and 0.91(t, 3H). Consistent with the expected results, the characteristic peaks of 4-PCL and L-PCL were very similar, resulting in little change in the characteristic peaks. In addition, CDCl_3_ was acidic, which could replace the active H of –OH in the sample, so the ^1^H peak of –OH had disappeared. The encouraging results were that the linear structure of L-PCL was complete, which was not destroyed by hydrolysis. The results were consistent with those reported in the literature [26]. Furthermore, the formation of solid particles could be used as an indirect evidence to prove that the hydrolyzed product of 4-PCL was L-PCL. However, it still needed other testing methods such as FT-IR to prove the conclusions.

### 3.2. FT-IR Spectra of PCL

**Figure 2:**
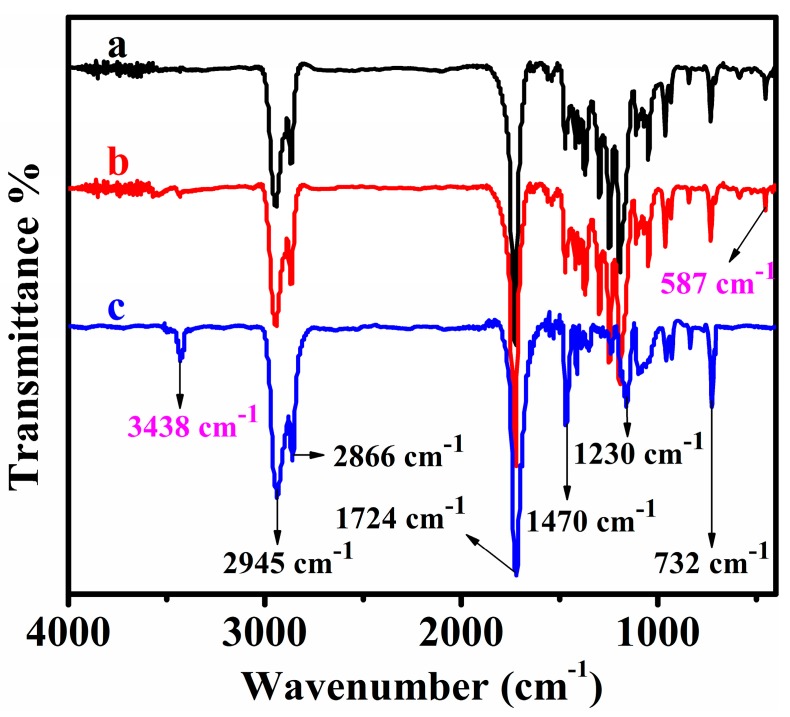
FT-IR spectra of product (**a**): 4-PCL; (**b**): 4-PCL treated with deionized water; (**c**): L-PCL.

Figure 2 showed the FTIR spectra of the 4-PCL and which were precipitated in distilled water and 10 *v*/*v* % HCl systems. It was found in Figure 2a that the absorption peaks of the –C=O group and –C–O–C– group were observed at 1724 cm^−1^ and 1240 cm^−1^. The absorption peaks at 2945 cm^−1^ and 2866 cm^−1^ were the peaks of the –CH_3_, –CH_2_– group. The absorption peaks of the –C–H was located at 1470 cm^−1^. In addition, the absorption peak of the Ti–O group was observed at 732 cm^−1^. Figure 2b showed there were no significant changes in the characteristic peaks during precipitation in distilled water. That phenomenon indicated that the polymers were not hydrolyzed under that condition. However, the interesting phenomenon was observed in Figure 2c. The new absorption peak of the –OH at 3438 cm^−1^ and the absence peaks of the Ti–O at 587 cm^−1^ indicated that 4-PCL may hydrolyze to single-chain PCL and TiO_2_ when precipitated in 10 *v*/*v* % HCl systems. Unlike initiator of TBT, 4-PCL was proved to keep stable in distilled water, the hydrolysis reaction could only take place under the acid-catalysis of HCI. With the cleavage of the Ti–O bond of Ti(OPCL)_4_, the L-PCL was obtained. According to the difference in thermal stability between 4-PCL and L-PCL, TG was used in the study of the thermal stability of polymers.

### 3.3. TG of PCL

**Figure 3:**
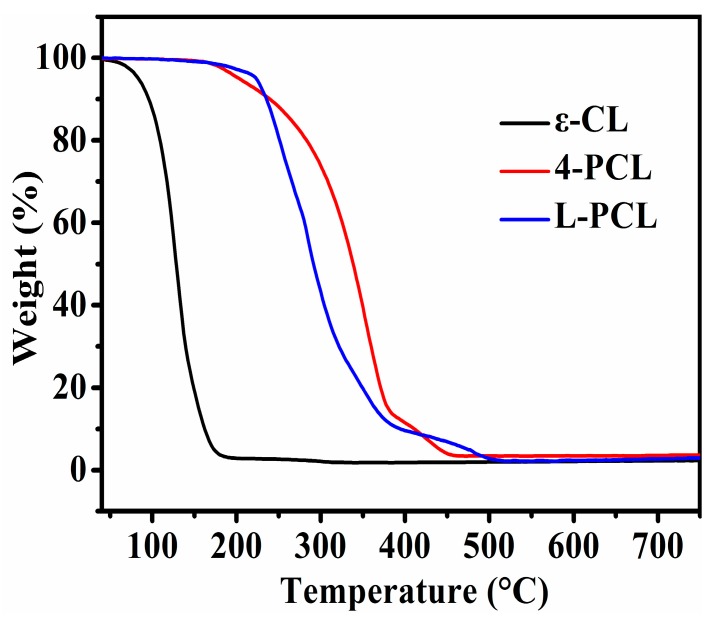
The TG curves of ε-CL, 4-PCL and L-PCL.

The TG curve of ε-CL, L-PCL and 4-PCL exhibited the effects of polymer architecture on the thermal stability. The test results were shown in Figure 3. Compared with the lower decomposition temperature of ε-CL at 100.9 °C, the initial degradation of 4-PCL was 160 °C whereas that for L-PCL degradation from 180 °C. It was noted that 4-PCL degraded over a much narrower temperature range than that of L-PCL. The shift of 4-PCL to lower temperature was due to the decomposing of the Ti–O bong. The degradation of PCL was ascribed to random cleavage through cis-elimination and specific chain end scission by unzipping from the hydroxyl end of the polymer chain. 4-PCL had many more ester chains than that of L-PCL, leading to greater unzipping behavior and, therefore, a faster degradation process [27]. In addition, thermal decomposition of the 4-PCL produced TiO_2_ that was the reason why there was still about 3 wt. % residual weight in the TG curve of 4-PCL.

### 3.4. SEM of TiO_2_

100 mg product and commercial TiO_2_ were respectively added into 20 g anhydrous ethanol with ultrasonic treatment for 30 min, and a small amount of the upper layer solution were dropped on the silicon wafer. Then the anhydrous ethanol was dried slowly by a blower. The silicon wafer was adhered to the conductive adhesive for SEM.

**Figure 4:**
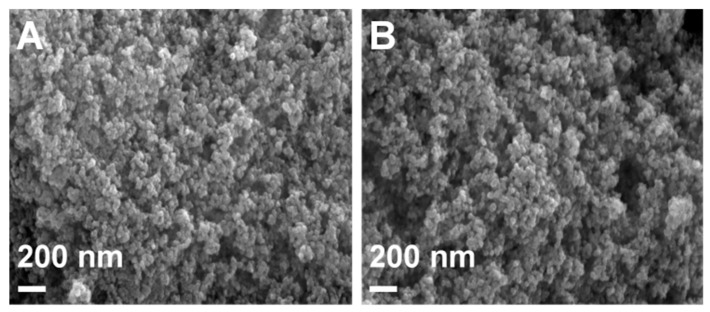
SEM images of (**A**) product TiO_2_ compared with (**B**) commercial TiO_2_.

The morphologies of samples were visualized by SEM. As can be seen in Figure 4, the hydrolyzed TiO_2_ exhibited a uniform spherical structure with an average size of about 20–30 nm. Compared with initiator of TBT, which was easily hydrolyzed to produce larger irregular particles, the hydrolytic stability of 4-PCL had been significantly enhanced. As an important solid anti-wear agent, TiO_2_ had been widely studied and applied in the lubrication field [28]. As the by-product, TiO_2_ could be added into the target product of synthetic base oil, which formed a nano-lubricant with excellent anti-wear performance.

### 3.5. XRD of TiO_2_

**Figure 5:**
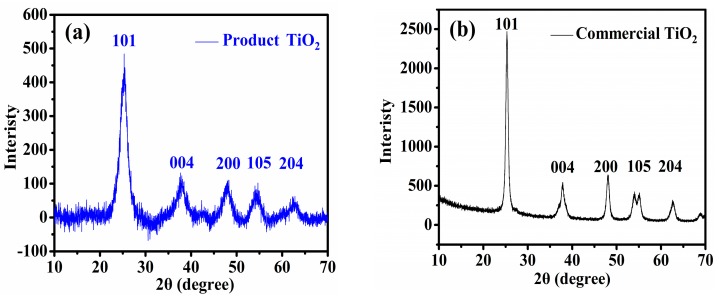
XRD pattern of product TiO_2_ (**a**) compared with commercial TiO_2_ (**b**).

The crystalline structure of TiO_2_ was analyzed, comparing with commercial TiO_2_. It could be observed from Figure 5 that the characteristic diffraction peaks of TiO_2_ at 25.23° (101), 37.77° (004), 47.97° (200), 54.35° (105) and 62.4° (204) were indexed to anatase phase. Compared with other crystalline, TiO_2_ of anatase type had the minimum relative density. Therefore, it was less prone to precipitation in the oil phase that played an important role in the dispersion stability of TiO_2_ in base oil. Anatase TiO_2_ with moderate hardness could form stable balls on the metal surface in lubrication system, which could play an important role in reducing wear. In addition, TiO_2_ had large dielectric constant and thermal conductivity, which enhanced the insulating ability and heat dissipation capability of the base oil.

However, the by-product TiO_2_ had a low crystallinity compared to commercial grade TiO_2_. It may be due to the good stability of long-chain PCL in the hydrolysis process, preventing part of the long-chain from undergoing hydrolysis and causing it to remain on. As an anti-wear additive, surface modification was usually required to improve the dispersion stability of nanoparticles in organic phase. If the phenomenon of low crystallinity of the by-product TiO_2_ was indeed caused by long chain molecules on the surface, it will undoubtedly play a positive role in the dispersion of the product TiO_2_ in the oil phase.

### 3.6. FT-IR Spectra of TiO_2_

**Figure 6:**
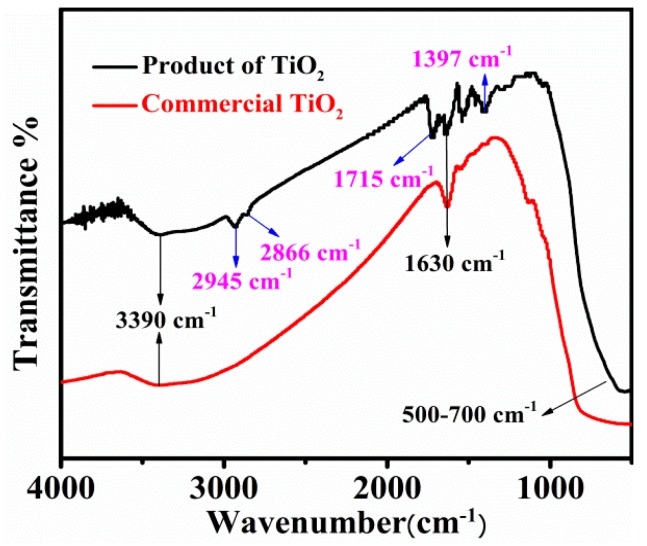
FT-IR spectra of product TiO_2_ and commercial TiO_2._

FTIR spectra of product nano-TiO_2_ comparing with commercially available TiO_2_ were shown in Figure 6. The characteristic peak of TiO_2_ was observed at about 500–700 cm^−1^. The absorption peaks at 3390 cm^−1^ and 1630 cm^−1^ were the –OH group from the crystal water adsorbed on the surface of TiO_2_. The new absorption peak at 2945 cm^−1^ and 2866 cm^−1^ corresponded to the –CH_2_–CH_2_– group. The absorption peaks of the –C–H and –C–H_3_ were located at 1397 cm^−1^ and 732 cm^−1^. The new absorption peak at 1715 cm^−1^ were the peaks of the –C=O group. The experimental results showed that the surface of nano-TiO_2_ dioxide had a long-chain ester bond. It was due to the high reactivity of TBT, the four-arm embedded chain length was inconsistent during the initiation of ε-CL ring-opening to form 4-PCL. Long-chain arms had proved to be more stable, resulting in the four-armed star-type PCL not completely forming L-PCL in the process of hydrolysis. Nano-TiO_2_ with ester chain had better compatibility in base oil, which could improve the dispersion stability of nanoparticles in base oil.

### 3.7. TG of TiO_2_

**Figure 7:**
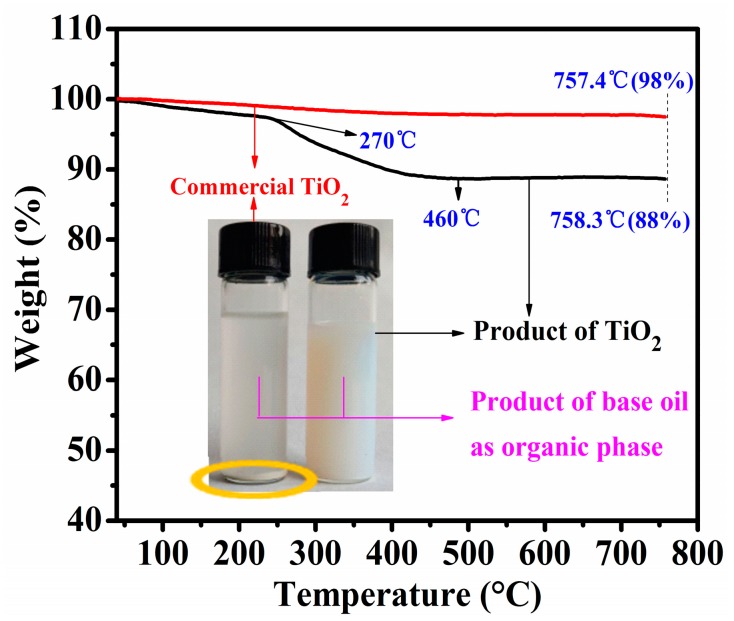
The TG curves of TiO_2_ and dispersion stability test of TiO_2_ (0.1 wt. %) 60 days after sonication.

The thermal behavior of TiO_2_ was investigated under an airflow rate of 50 mL/min. The temperature scans were set in the range from 40 to 800 °C with an increasing rate of 10 °C /min. The TG curve of TiO_2_ comparing with the commercial TiO_2_ were shown in Figure 7. The initial slope in the degradation profiles of TiO_2_ could be attributed to the water loss from the sample in the temperature ranging from 40 °C to 270 °C. The main mass loss of TiO_2_ was 11% from 270 °C to 460 °C, which was due to the decomposition of long-chain ester groups on TiO_2_. The temperature curve of decomposition was consistent with that of 4-PCL, which provided a basis for the assumption. The experimental results showed that the TBT initiator had high activity, which could initiate the ring-opening polymerization of ε-CL in a short time, resulting in uneven four-arm length. As the stability of Ti–O was enhanced with the increase of arm length, there were still a small number of linear long-chain ester chains on the surface of TiO_2_. The long ester chain could help to increase the compatibility of TiO_2_ in the base oil. It was very important to improve the dispersion stability of nano-TiO_2_ in base oil. Figure 7 also provided a qualitative assessment of the performance of dispersion stability for product nano-TiO_2_ and commercially available TiO_2_ in the base oil. After ultrasonic treatment with 0.1 wt.% nano-TiO_2_, the product nano-lubricant could be stable and no precipitation phenomenon was observed during 60 days. On the contrary, the commercially available TiO_2_ were obviously deposited after seven days.

### 3.8. FT-IR Spectra of Modified TMT

**Figure 8:**
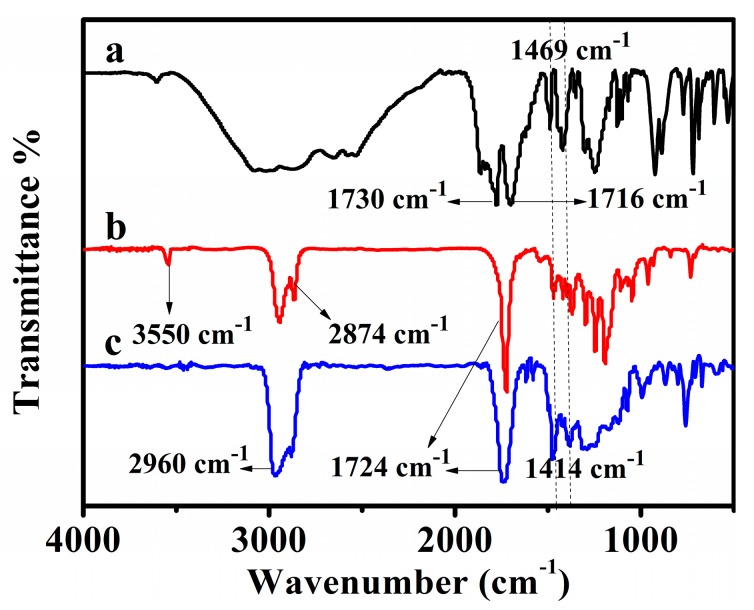
FT-IR spectra of (**a**) TMA, (**b**) polymer intermediates and (**c**) the product of base oil.

FT-IR spectra of TMA, polymer intermediates and the product of base oil were shown in Figure 8. It was found in Figure 8a that the absorption peaks of the –C=O groups were observed at 1730 cm^−1^ and 1716 cm^−1^, which were the characteristic peaks of TMA. The characteristic absorption of benzene ring appeared at 1469 cm^−1^ and 1414 cm^−1^. Figure 8b showed that the two characteristic peaks corresponding to –C=O groups at 1730 cm^−1^ and 1716 cm^−1^. Correspondingly, a new –C=O peak was formed at 1724 cm^−1^. The absorption peaks at 2945 cm^−1^ and 2866 cm^−1^ were the peaks of the –CH_2_–CH_2_– group. The absorption peaks at 3500 cm^−1^ was the –COOH group of polymer intermediates. Compared with Figure 8b, the absorption peaks of the –C–H and –CH_3_ were located at 1470 cm^−1^ and 732 cm^−1^. In addition, the absence of an –OH peak at 3550 cm^−1^ conformed that the reaction of TMA with L-PCL had been reacted completely, leading to residual –OH groups. It was also certain that the esterification reaction between the –COOH groups of the polymer intermediates and isobutanol was complete. Both of the reactions led to the complete absence of peak at 3550 cm^−1^. The results showed that the reaction process was thorough and the infrared spectra were consistent with the target products.

### 3.9. ^1^H-NMR Spectra of Modified TMT

**Figure 9:**
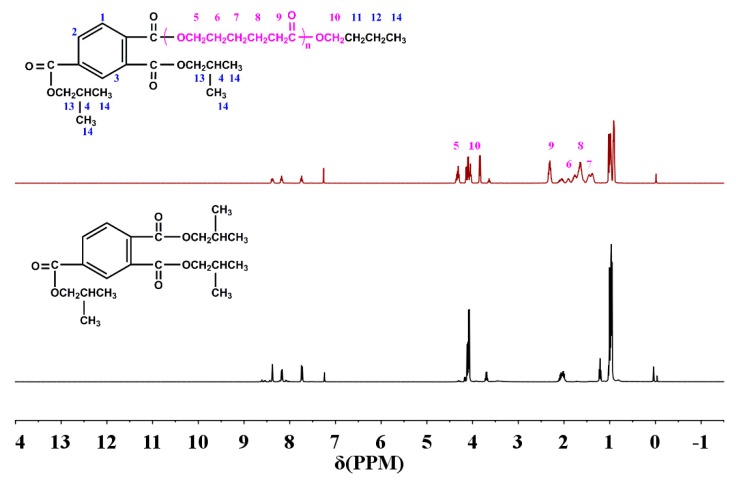
^1^H-NMR spectra of TMT.

Figure 9 showed a typical ^1^H-NMR spectrum of the product of modified TMT in comparison with unmodified TMT. The peaks at δ: 8.38(s, 1H), 8.18(s, 1H) and 7.79(s, 1H) were attributed to the protons of benzene ring. The key changing characteristic ^1^H peaks of protons could be observed at δ: 4.31(t, 2H), 2.31(t, 2H) and 4.05(t, 2H). The results demonstrated that the product structure was consistent with the design structure. In addition, the insertion of PCL into the structure of the TMT was successfully achieved. MS analysis was carried out by electrospray ionization (ESI). The relative abundance was 100% of the mass spectrum peak *m*/*z*. The characteristic peak was attributed to ESI-MS *m*/*z* 835.4841 [M+H^+^] and 857.4709 [M+Na^+^], which were consistent with the theoretical value of 834.4766. Experimental result proved that the synthetic route was successful.

### 3.10. Effect of Embedded Chain Length on TMT Performance

#### 3.10.1. Effect of Embedded Chain Length on Lubrication Performance of TMT

The application of TMT lubricants had been greatly limited due to the low viscosity index. How to improve the viscosity index and expand its application in lubrication had attracted wide attention. The experimental results in Table 1 showed that the embedding of PCL into the structure of TMT could effectively increase the viscosity index of base oil, and the viscosity index increased with the increase of the embedded content. Compared with unmodified TMT, the viscosity index of the modified TMT increased from 8 to above 100, which greatly improved its viscosity–temperature performance. The soft segments of embedded PCL greatly enhanced the ability of the base oil to resist the effects of temperature. In addition, as the length of the embedded chain increased, the kinematic viscosity first decreased and then increased. The grown chain increased the intermolecular distance, which lead to a decrease in intermolecular force and kinematic viscosity. When the chain was long enough to cause entanglement, the viscosity increased sharply. With the increase of flexible groups in the modified base oil, the elastic properties of the oil molecules were improved, which was reflected in increase of viscosity index and the improvement of low temperature properties. However, as a semi-crystalline polymer, the crystallization ability of the polymer increased as the content of PCL increased, and once the polymer was crystallized, its softness was greatly lowered, resulting in a decrease in its low-temperature fluidity. Just like a mop placed outdoors in winter, once it freezes, its elastic properties dropped rapidly. Acid value (AV) could be used as the basis for judging the degree of esterification. The experimental results show that the reaction was thorough and the AV can meet the requirements of lube base oil. 

#### 3.10.2. Effect of Embedded Chain Length on Thermal Stability Performance of TMT

**Figure 10:**
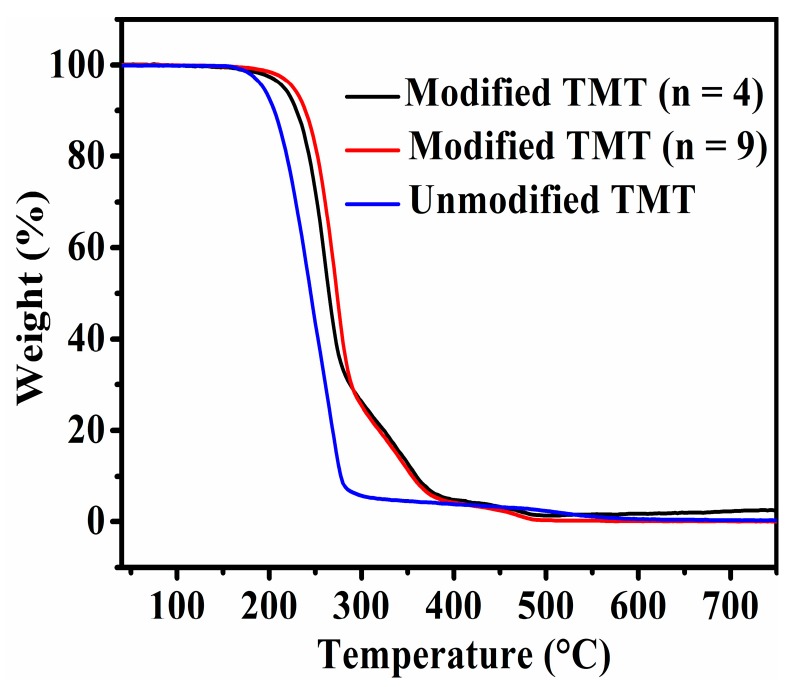
TG curves of modified TMT with different chain length comparing with unmodified TMT (*n*: average embedded chain length of PCL).

Comparing with the lower decomposition temperature of unmodified TMT of 202.5 °C, the TG curve of product with n = 4 and n = 9 exhibited the effects of embedded chain length on the thermal stability in Figure 10. The initial degradation of base oil with lower chain length began from 237.2 °C to 480 °C whereas for base oil with longer chain length degradation began from 246.7 °C to 480 °C. The experimental results showed that the thermal stability of the base oil could be improved with the increase of the embedded chain length. Notably, this was consistent with the previous experimental conclusions.

#### 3.10.3. Effect of Embedded Chain Length on Anti-Wear Property of TMT

**Figure 11:**
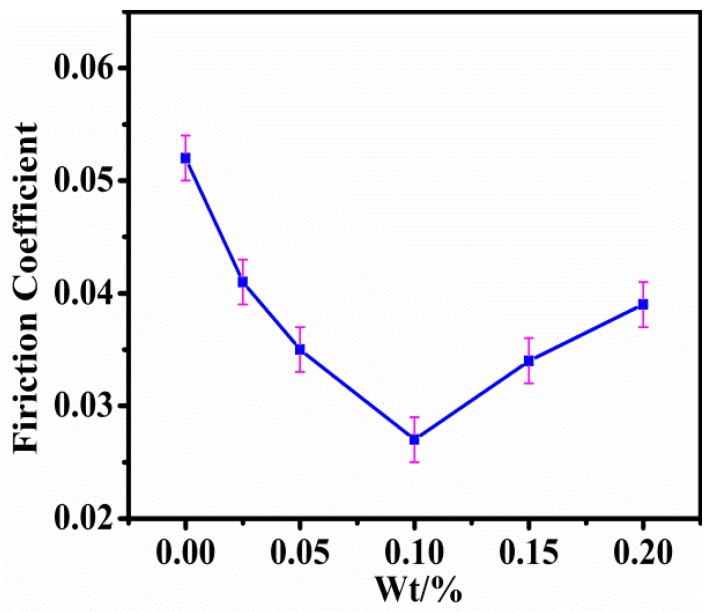
Friction coefficient lubricating by product TiO_2_ containing oil at different concentrations.

The modified TMT (n = 4) was used for the evaluation of the anti-wear property and the influence of the raw material alcohol on the lubricating property of the base oil. Figure 11 illustrated the variation of friction coefficient with nano-TiO_2_ content for modified TMT lubricant base oil under a load of 588 N for 60 min. It could be inferred from Figure 11 that, when the content of nano-TiO_2_ in lubricant was below 0.1%, the friction coefficient reduced sharply until attaining a minimum value with the concentration of nanoparticles. The friction coefficients with nano-TiO_2_ adding were changed from 0.052 to 0.027, which reduced by 48.1% compared with base oil. Afterwards, increasing the nano-TiO_2_ had a reverse effect on friction reduction of modified TME base oil, which led to a gradual increase of the friction coefficient. The result implied that a certain optimal concentration of nano-TiO_2_ could provide excellent friction-reduction and anti-wear ability of pure lubricant significantly. Particularly, even at concentrations exceeding the optimal amount, the performance of modified nano-lubricant base oil was considerably much better than the base oil.

**Figure 12:**
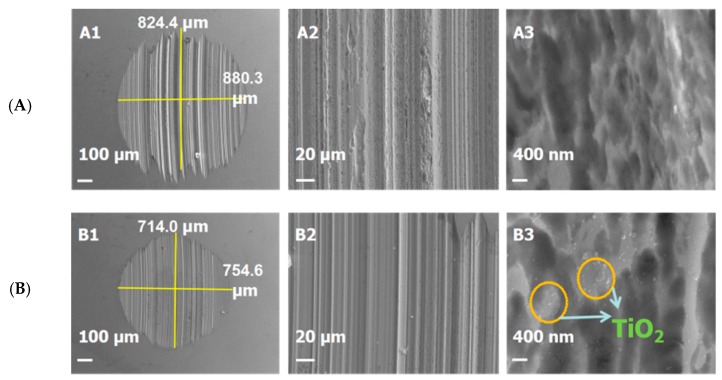
SEM of surface lubricated with product base oil (**A**) and product TiO_2_ nano-lubricant (**B**).

Figure 12 showed the morphologies of the worn surface lubricated by modified TMT and modified product TiO_2_-containing TMT under a load of 588 N for 60 min.

**Scheme 2:**
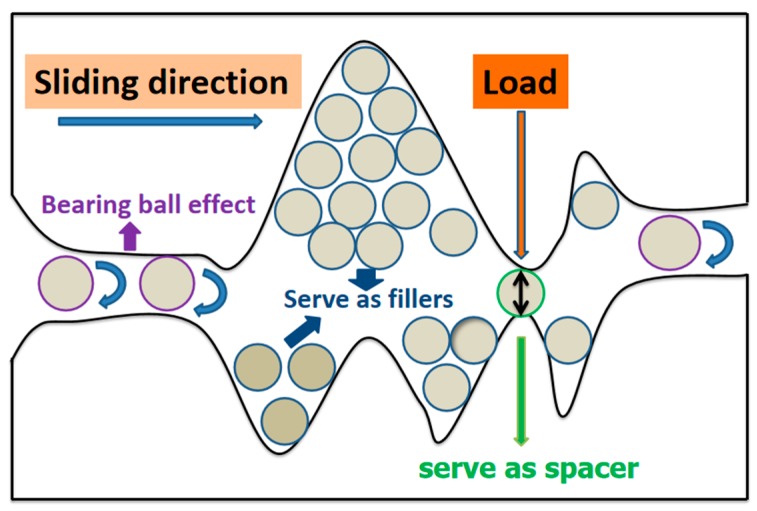
Anti-wear mechanismatic of nano-TiO_2._

Comparing with base oil (A), under the lubrication of TiO_2_-containing base oil (B), the worn surfaces were much smoother and smaller in size. Average wear spot diameter was reduced from 852.4 μm to 734.3 μm. It could be observed from B3 that friction surfaces were filled with TiO_2_, which not only make up the surface abrasion, but also optimize the surface geometry roughness, so the overall friction coefficient and abrasion loss were relatively low. The widely accepted anti-wear theories were ball effect, filling and repairing effect and third body protection [29,30,31]. Nano-TiO_2_ had the effect of reducing the friction coefficient, decreasing abrasion and repairing wear defects [32]. The friction characteristics of base oil had been improved. The anti-wear mechanism of nano-TiO_2_ was shown in Scheme 2.

#### 3.10.4. Effect of Raw Alcohol on Lubrication Property of Modified TMT

The experimental results of Table 2 showed that as the carbon chain of the raw material alcohol increased, the kinematic viscosity and the viscosity index of the product oil gradually increased. Especially at high viscosity, the viscosity index can still be steadily increased, which was very important for the application of product oil in high temperature chain oil and metal cutting fluid.

## 4. Conclusions

In this article, TMT was upgraded via embedding PCL into base oil structure to solve the problem of the low viscosity index. Compared with unmodified TMT, with the addition of PCL embedded chain, the viscosity index of the base oil increased from 8 to above 100, and the viscosity index reached the high viscosity index lubricating oil level (80–100), which even satisfies special high-grade viscosity index lubricant index (above 110). With the increase of the length of the embedded chain, the thermal stability of the modified base oil can be greatly improved. In addition, since the PCL had good compatibility, the biodegradability of the base oil can be improved to meet the requirements of the green lubricating oil. As the only by-product, nano-TiO_2_ was incompletely hydrolyzed, and the surface residual polyester chain provided assistance for the stable dispersion of nano-TiO_2_ in the organic phase. The significant advantage was to avoid the requirement for surface modification of nanoparticles in the process of compounding nano-lubricants. It also provided a new idea for the experimental route of surface modification of nano-materials. The anti-wear performance of the lubricating system was optimized by combining the by-product nano-TiO_2_ with modified TMT. More importantly, this experiment provided new ideas for improving the performance of synthetic lubricants.

## Figures and Tables

**Table 1:** Effect of embedded chain length on TMT lubrication performance.

Property	TMT (n: AVERAGE Embedded Chain Length of PCL)						
	n = 0	n = 3	n = 4	n = 5	n = 6	n = 7	n = 9
AV (mg KOH/g)	1.08	1.01	1.04	0.98	1.05	0.88	0.99
KV (40 °C, mm^2^/s)	80.91	78.45	58.55	51.06	50.22	128.0	131.0
KV (100 °C, mm^2^/s)	7.28	9.86	8.18	7.54	7.70	15.06	15.39
VI	8	105	107	110	119	120	121
PP (°C)	−45	−36	−35	−33	−33	−30	−27

AV: Acid number; KV: Kinematic viscosity; VI: Viscosity Index; PP: Pour point.

**Table 2:** Effect of raw alcohol on lubrication properties of TMT.

Property	Raw Alcohol		
	Isobutanol	Iso-Octanol	1-Tetradecanol
AV (mg KOH/g)	1.04	0.95	0.86
KV (40 °C, mm^2^/s)	58.55	68.77	452.8
KV (100 °C, mm^2^/s)	8.18	9.35	42.3
VI	107	114	145
PP (°C)	−35	−35	−4

AV: Acid number; KV: Kinematic viscosity; VI: Viscosity Index; PP: Pour point.
